# The activity of the pyrrole insecticide chlorfenapyr in mosquito bioassay: towards a more rational testing and screening of non-neurotoxic insecticides for malaria vector control

**DOI:** 10.1186/s12936-015-0639-x

**Published:** 2015-03-24

**Authors:** Richard M Oxborough, Raphael N’Guessan, Rebecca Jones, Jovin Kitau, Corine Ngufor, David Malone, Franklin W Mosha, Mark W Rowland

**Affiliations:** Department of Disease Control, London School of Hygiene and Tropical Medicine, London, UK; Department of Entomology and Parasitology, Kilimanjaro Christian Medical University College, Moshi, Kilimanjaro Tanzania; CREC laboratories, Centre de Recherche Entomologique de Cotonou, Laboratoire Nationale, Ministère de la Santé, Cotonou 06, BP 2604, Benin; Pan-African Malaria Vector Research Consortium, (PAMVERC), Moshi, Tanzania; Innovative Vector Control Consortium (IVCC), Liverpool School of Tropical Medicine, Pembroke Place, Liverpool, L3 5QA UK

**Keywords:** Chlorfenapyr, ITN, *Anopheles gambiae*, Vector control, Bioassay, Insecticide, Malaria

## Abstract

**Background:**

The rapid selection of pyrethroid resistance throughout sub-Saharan Africa is a serious threat to malaria vector control. Chlorfenapyr is a pyrrole insecticide which shows no cross resistance to insecticide classes normally used for vector control and is effective on mosquito nets under experimental hut conditions. Unlike neurotoxic insecticides, chlorfenapyr owes its toxicity to disruption of metabolic pathways in mitochondria that enable cellular respiration. A series of experiments explored whether standard World Health Organization (WHO) guidelines for evaluation of long-lasting insecticidal nets, developed through testing of pyrethroid insecticides, are suitable for evaluation of non-neurotoxic insecticides.

**Methods:**

The efficacy of WHO recommended cone, cylinder and tunnel tests was compared for pyrethroids and chlorfenapyr. To establish bioassay exposure times predictive of insecticide-treated net (ITN) efficacy in experimental hut trials, standard three-minute bioassays of pyrethroid and chlorfenapyr ITNs were compared with longer exposures. Mosquito behaviour and response to chlorfenapyr ITN in bioassays conducted at night were compared to day and across a range of temperatures representative of highland and lowland transmission.

**Results:**

Standard three-minute bioassay of chlorfenapyr produced extremely low levels of mortality compared to pyrethroids. Thirty-minute day-time bioassay produced mortality closer to hut efficacy of chlorfenapyr ITN but still fell short of the WHO threshold. Overnight tunnel test with chlorfenapyr produced 100% mortality and exceeded the WHO threshold of 80%. The endogenous circadian activity rhythm of anophelines results in inactivity by day and raised metabolism and flight activity by night. A model which explains improved toxicity of chlorfenapyr ITN when tested at night, and during the day at higher ambient temperature, is that activation of chlorfenapyr and disruption of respiratory pathways is enhanced when the insect is more metabolically and behaviourally active.

**Conclusions:**

Testing according to current WHO guidelines is not suitable for certain types of non-neurotoxic insecticide which, although highly effective in field trials, would be overlooked at the screening stage of evaluation through bioassay. Testing methods must be tailored to the characteristics and mode of action of each insecticide class. The WHO tunnel test on night-active anophelines is the most reliable bioassay for identifying the toxicity of novel insecticides.

## Background

Owing to the evolution and selection of high-level resistance to pyrethroid insecticides in African malaria vectors, there is an urgent need to develop novel insecticides for mosquito net and indoor residual use [1-3]. The need for safe, alternative insecticides is particularly acute for mosquito nets [4], as no new insecticides have been recommended by World Health Organization (WHO) since pyrethroids were introduced in the 1980s [5,6]. In the search for new active ingredients it is essential that any biological screen of chemical toxicity is representative and does not deviate from levels of exposure experienced by vectors under natural (i.e., household) conditions, otherwise potential new classes of toxin might be easily overlooked. Current WHO guidelines for identifying new insecticides and measuring toxic activity against malaria vectors are based on historic precedents established for neurotoxins, such as pyrethroids, organochlorines, carbamates, and organophosphates [7,8]. The specific guidelines for insecticide-treated and long-lasting nets are firmly rooted on knowledge accumulated by the WHO Pesticide Evaluation Scheme (WHOPES) during the testing of fast-acting pyrethroid products [8]. The initial screen and assessment of insecticide efficacy is done using a WHO cone test in which mosquitoes are exposed to treated material for just three minutes and mortality recorded a day later [7]. This is adequate for most types of pyrethroid and will distinguish highly active from less toxic compounds [7]. However, this approach, using such short exposure times, may not be suitable for screening and identifying novel classes of insecticide if new classes of toxin do not excito-repel or act as fast as the pyrethroids.

Chlorfenapyr is an insecticide new to vector control from the class known as pyrroles [9,10]. Pyrroles are broad-spectrum insecticides, which show contact and stomach toxicity [11,12]. They are pro-insecticides which require initial activation by mixed function oxidases to produce the active compound [10]. Unlike the pyrethroids and all other classes of insecticide currently approved for adult mosquito control, the pyrroles’ site of action is not the insect nervous system. Instead, pyrroles act at the cellular level and disrupt respiratory pathways and proton gradients through the uncoupling of oxidative phosphorylation in mitochondria [10]. Because of its unique mode of action, chlorfenapyr shows no cross resistance to mechanisms that confer resistance to standard neurotoxic insecticides against the mosquitoes *Anopheles gambiae*, *Anopheles funestus* and *Culex quinquefasciatus* [13,14], bed bugs *Cimex* spp. [15,16], or beet armyworm *Spodoptera exigua* [17]. When applied to mosquito nets occupied by human volunteers in experimental hut trials, chlorfenapyr induces relatively high rates of mortality among host-seeking mosquitoes regardless of their pyrethroid resistance status [18,19]. Yet in some types of laboratory bioassay, chlorfenapyr appears slow acting or induces patterns or levels of mortality that are not typical of neurotoxic insecticides and not predictive of mortality induced by chlorfenapyr-treated nets in hut trials [11,18]. Since chlorfenapyr is both activated by and acts upon oxidative/respiratory pathways, its toxicity may be especially sensitive to temperature or to the physiological status of the insect, which in the case of the anopheline mosquito is more metabolically active by night than by day due to the phase of their circadian rhythm [20,21]. A new, long-lasting, insecticide-treated net based on chlorfenapyr is being developed commercially. As part of the development process the properties and toxicity of chlorfenapyr were explored using a range of bioassay systems under ambient and controlled conditions in order to better understand the mode of action of pyrroles and to develop assay systems more appropriate for screening and evaluating non-neurotoxic insecticides.

The need to modify bioassay techniques for evaluation of novel classes of LLIN insecticides is recognised as a possibility in the latest WHOPES LLIN guidelines [8]. In the series of experiments presented, chlorfenapyr serves as representative novel insecticide and pathfinder for a more rational approach for the determination of chemical toxicity and bioassay thresholds that are more predictive of activity under field conditions.

## Methods

### Insecticide formulations

Bioassay testing were carried out in parallel at the two Pan-African Malaria Vector Research Consortium (PAMVERC) trial sites in Moshi, Tanzania, and Cotonou, Benin, during the course of a project between BASF and Innovative Vector Control Consortium (IVCC) aimed at developing a novel type of LLIN. Comparison is made between chlorfenapyr and the pyrethroid alphacypermethrin which serves as a positive control. Polyester netting, 100-denier, was treated with chlorfenapyr suspension concentrate (SC) 214.5 g/l, (BASF, Ludwigshafen, Germany) or alphacypermethrin SC 60 g/l (BASF, Ludwigshafen, Germany). Each batch of chlorfenapyr insecticide-treated net (ITN) was tested in Ludwigshafen, Germany using gas–liquid chromatography to confirm that mean dosages were within 10% of target. Chlorfenapyr ITN samples are described as “with” or “without binder” depending on whether polymers were added to the SC formulation to improve wash resistance. While the dosages applied, and adjuvants added differed during product development, all experiments investigating the effects of external factors or conditions were carefully controlled or adjusted for in the statistical analysis.

### Testing overview

In the first series, the mosquito mortality generated in Phase 2 experimental hut trials of treated nets was calibrated against mortality generated in Phase 1 bioassay tests in an attempt to determine more realistic bioassay exposure times. In the second series, the standard WHOPES bioassay tests (cone bioassay, cylinder bioassay, tunnel test) and the efficacy thresholds established for the pyrethroid class were assessed for their suitability for pyrroles. In the third, mosquito circadian motor activity in bioassay chambers was compared by day and by night. In the fourth, the response to insecticide in cone bioassay was compared by day and by night. In the fifth, the response to insecticide was compared across a range of temperatures representative of highland and lowland transmission.

### Determining rational exposure times for contact bioassay more predictive of response in field conditions

The primary objective was to determine whether percentage mortality achieved using WHOPES standard three-minute contact bioassay was a fitting predictor of chlorfenapyr ITN field performance or whether exposure time should be changed. This was demonstrated by comparing mortality in bioassay with mortality of wild free-flying *Anopheles arabiensis* in experimental hut trials in Tanzania*.* The methodology and results of the trial (mortality and blood-feeding inhibition) have been published previously [14]. Hand-dipped mosquito nets treated with chlorfenapyr 100 mg/sq m or alphacypermethrin 25 mg/sq m were tested in the experimental huts for four weeks. All ITNs used in the trial were tested in wire-ball frame bioassays two days before the trial started to assess toxicity against F1 generation of wild-caught *An. arabiensis* that were resistant to pyrethroids [22,23]. Testing methodology was based on WHO protocol [7] with the standard three-minute exposure compared against a prolonged 30-minute exposure. Mortality was recorded after 24, 48 and 72 hours to assess any delayed mortality which is consistent with the mode of action of chlorfenapyr [11]. Cotton pads soaked with 10% glucose were provided throughout (and for all subsequent tests unless stated otherwise).

### Efficacy of chlorfenapyr compared to alphacypermethrin in standard contact bioassay and tunnel tests

The standard WHOPES bioassay tests (cone bioassay, cylinder bioassay, tunnel test) and the efficacy thresholds established for pyrethroids were assessed for their suitability for chlorfenapyr [8]. Day-time cone and cylinder bioassays with the standard three-minute exposure were compared with a prolonged 30-minute exposure. After testing, mosquitoes were transferred to controlled temperature incubators (LMS Models 240 and 600, Sevenoaks, UK) and held at 27°C ± 0.5°C. Tunnel tests were conducted according to WHOPES protocol using the same netting samples and test conditions [8]. The netting treatments tested were chlorfenapyr 200 mg/sq m and alphacypermethrin 25 mg/sq m. Testing was done in Benin using insecticide susceptible *An. gambiae* Kisumu.

### Mosquito circadian activity in bioassay chambers during day and night phases

The objective was to observe mosquito behaviour, flight and resting activity, in chambers of similar size to WHO cones and cylinders and compare this during day-time and night-time hours. The activity of mosquitoes was monitored continuously using an acoustic actograph, attuned to the wing-beat frequency of flying mosquitoes, and which detects the spontaneous take-offs and landings of individual mosquitoes without need for external interference or stimulation [20,21]. Twenty-four recording chambers were constructed from standard 250-ml reagent bottles that had their glass bases removed and with each chamber separated from its microphone by a polyethylene membrane fitted to the base of the reagent bottle. Individual mosquitoes were housed in each chamber and provided with a small tubule of sugar solution. The output from each microphone fed into circuit that amplified the wing-beat signals and operated the relay of an event-recorded pen. Each mosquito was given a score of 1 for any minute that contained flight activity, and thus a total of between 0 and 60 for each hour. These activity scores were averaged and used to produce histograms of hourly activity against time. *Anopheles stephensi* females were tested at five to six days of age, and were inseminated, and sugar-fed rather than blood-fed, consistent with host-seeking mosquitoes. Testing was done using groups of 24 females over a period of four to five days. In the first experiment, females were recorded in a 12-hour light phase and 12-hour dark phase (LD 12:12) synchronized with the insectary rearing regime. In the second experiment females were recorded in constant darkness (LD 0:24).

### Insecticide bioassay efficacy related to the phase of the mosquito circadian rhythm

The aim of this study was to determine whether exposure to chlorfenapyr ITN in bioassay as done normally during the day-time (12-hour light from 07:00–19:00) produced a different mortality response than testing during the night-time (12-hour dark from 19:00–07:00) phase when anophelines are inherently more active metabolically and behaviourally due to the phase of their circadian rhythm [20,21]. Cylinder bioassays with 30-minute exposure were conducted in Tanzania and Benin comparing testing in the day-time between 10:00 and 16:00 and in the night-time between 19:00 and 23:00. Mosquitoes were taken from the same population cohort, and divided into one group for night-time testing and one for day-time testing. The insectary and incubator were set to a LD 12:12 cycle from 07:00–19:00. Lights were kept off during dark phase testing and kept on during day phase testing. Testing and 72-hour holding conditions were set at 27°C ± 0.5°C with relative humidity (RH) 75% ± 15%. Three series of tests were done, two in Tanzania and one in Benin. In the first Tanzanian series, seven replicate netting samples were treated in Germany with 200 mg/sq m chlorfenapyr without binder. Testing was with *An. gambiae* Kisumu (pyrethroid susceptible). In the second Tanzanian series, five netting samples were treated in Germany with 200 mg/sq m chlorfenapyr plus binder. Testing was with *An. arabiensis* F1. In the Benin series, the same five netting samples treated in Germany with chlorfenapyr 200 mg/sq m plus binder were tested against pyrethroid resistant *An. gambiae* VK-PER.

### Effect of temperature on bioassay efficacy

The aim of this study was to determine whether the response to chlorfenapyr was dependent on ambient temperate during testing in day-time cylinder bioassays. In the first series, the 1-hour acclimation (pre-exposure), 30-minute insecticide exposure and 72-hour post-exposure holding was conducted at 22°C ± 1°C and 27°C ± 1°C using thermostatically controlled insectary convection heaters and air conditioners. *Anopheles gambiae* Kisumu (pyrethroid susceptible) was exposed for 30 minutes to seven replicate netting samples of 200 mg/sq m chlorfenapyr without binder and mortality recorded at 24-hour intervals up to 72 hours.

In the second series, cylinder tests were conducted at 2°C intervals between 21-29°C. After exposure at the required temperature mosquitoes were transferred to incubators set to the same testing temperature ±0.5°C and 75% ± 20% RH for 72 hours holding. Temperature and humidity were monitored using calibrated data loggers (Gemini tinytag TV-4500, West Sussex, UK). Netting treatments were with chlorfenapyr and alphacypermethrin. Testing at different temperatures had to be done sequentially rather than in parallel due to the limited number of incubators available.

### Analysis

#### Timing of bioassay in relation to mosquito circadian rhythm

Mixed effect logistic regression models were used to model mortality separately in each species or strain of mosquito (*An. gambiae* Kisumu, *An. arabiensis* F1 and *An. gambiae* VKPER) using STATA 10 software (STATA Corp, College Station, USA). All statistical modelling was performed on the log odds scale at the individual mosquito level with a random effect specified to account for similarities in mosquitoes tested at the same time point, and for potential behavioural clustering within the same test batch. The main predictor of interest was time of testing (night *vs* day). Statistical models additionally adjusted for insecticide, washing status, treatment technique, drying temperature, and interactions between each of these covariates and time of testing. The initial model for each species was simplified by removing each interaction term in turn via a process of manual backwards elimination until only simple covariates and statistically significant (p = 0.05) interactions with time of day remained.

### Effect of temperature on bioassay efficacy

Mixed effect logistic regression models were used as above. The main predictor of interest was testing temperature. For the 22°C *versus* 27°C comparison, **s**tatistical models additionally adjusted for country where testing was done (Benin or Tanzania) and treatment preparation. For the 21-29°C testing range the same modelling was performed but adjusted for insecticide (chlorfenapyr or alphacypermethrin).

### Ethical approval

Ethical approval was granted from the Tanzania National Institute of Medical Research (NIMR/HQ/R.8c/Vol.I/24) and London School of Hygiene & Tropical Medicine Ethics Committee (Application no. 5162).

## Results

### Determining rational exposure times for contact bioassay predictive of exposure to ITN under field conditions

**Figure 1:**
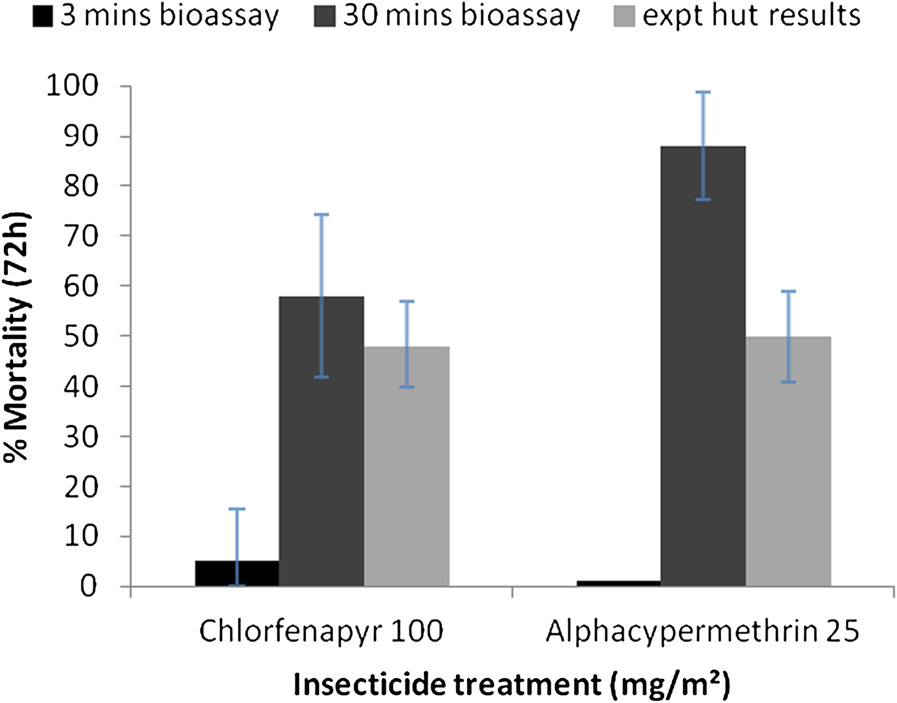
**Comparison of experimental hut mortality of free flying wild** ***Anopheles arabiensis*** **mosquitoes in the presence of occupied ITNs and ball bioassay mortality after three and 30 minutes exposure to the same ITNs (see [** 8 **]).**

Three-minute ball bioassay with 100 mg/sq m chlorfenapyr-treated netting resulted in mortality of only 5% against F1 wild *An. arabiensis*, compared to 48% in experimental hut trials of chlorfenapyr-treated nets against wild, free-flying *An. arabiensis* (Figure 1). Clearly, three minutes exposure in bioassay failed to predict performance against host-seeking mosquitoes in huts. Prolonged exposure of 30 minutes resulted in 58% mortality, closer to the mortality of free-flying mosquitoes. Mortality of pyrethroid-resistant F1 *An. arabiensis* [22,23] was also low for the alphacypermethrin netting in three-minute ball bioassay (1%) but the alphacypermethrin-treated nets were effective in experimental hut trials and killed 50%. Prolonged exposure of *An. arabiensis* to alphacypermethrin netting in bioassay (30 minutes) killed 88%.

### Efficacy of chlorfenapyr compared to alphacypermethrin in standard contact bioassay and tunnel tests

**Figure 2:**
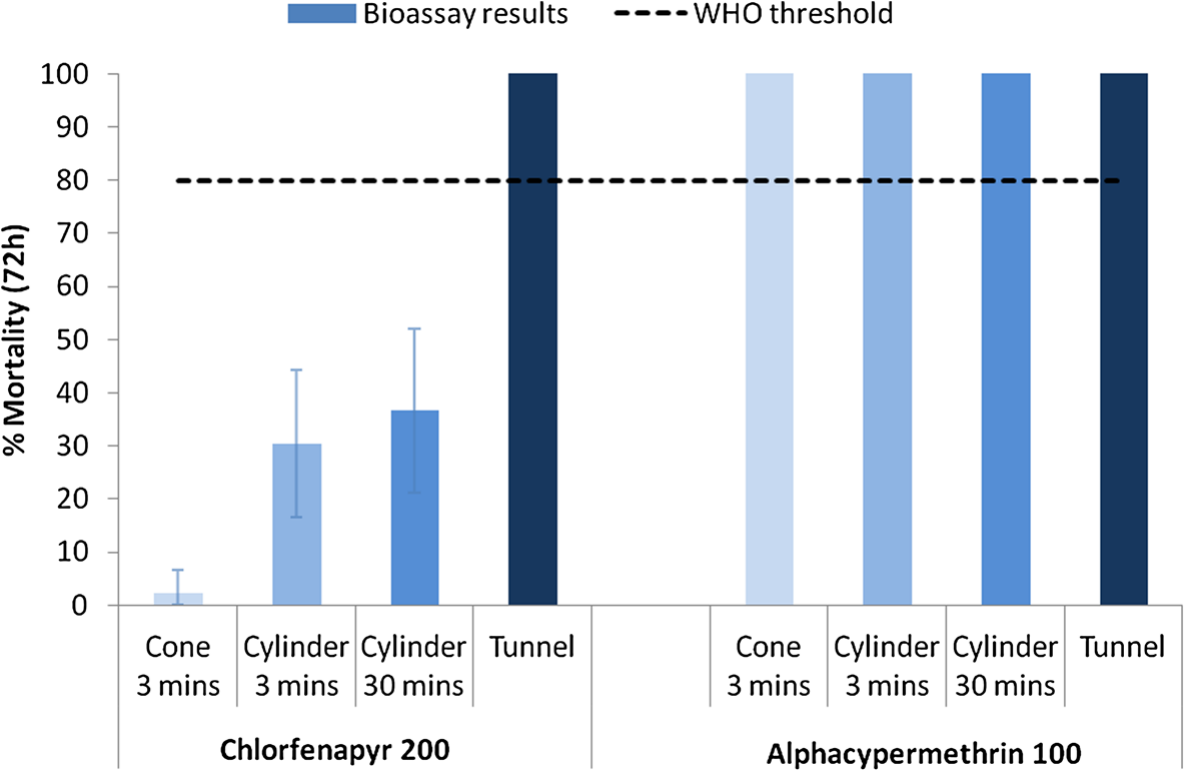
**Comparison of bioassay response in** ***Anopheles gambiae*** **Kisumu to chlorfenapyr and alphacypermethrin-treated nets using standard WHO bioassay techniques: day-time cone and cylinder bioassays and night-time tunnel tests.**

Under laboratory conditions a standard three-minute cone bioassay on chlorfenapyr ITN 200 mg/sq m produced <5% mortality, while three-minute exposure to the same chlorfenapyr netting in cylinder tests killed 30%. More prolonged, 30-minute exposure in cylinder tests produced 37% mortality. When tested in overnight tunnel tests, mortality was far greater reaching 100% (Figure 2). Adopting the WHO success threshold of 80% mortality in cone or cylinder bioassay, chlorfenapyr failed to meet this criterion with the standard three-minute exposure. Not even 30 minutes exposure was sufficient to reach 80% mortality. But chlorfenapyr did reach the 80% threshold using the tunnel test. By contrast, the 25 mg/sq m alphacypermethrin netting produced 100% mortality of susceptible *An. gambiae* Kisumu in cone and cylinder tests with three-minute exposure. Alphacypermethrin therefore met the WHO success threshold of 80% within the standard three-minute exposure and therefore did not need to undergo tunnel testing to achieve this criterion.

### Mosquito circadian behaviour in bioassay chambers during day and night

**Figure 3:**
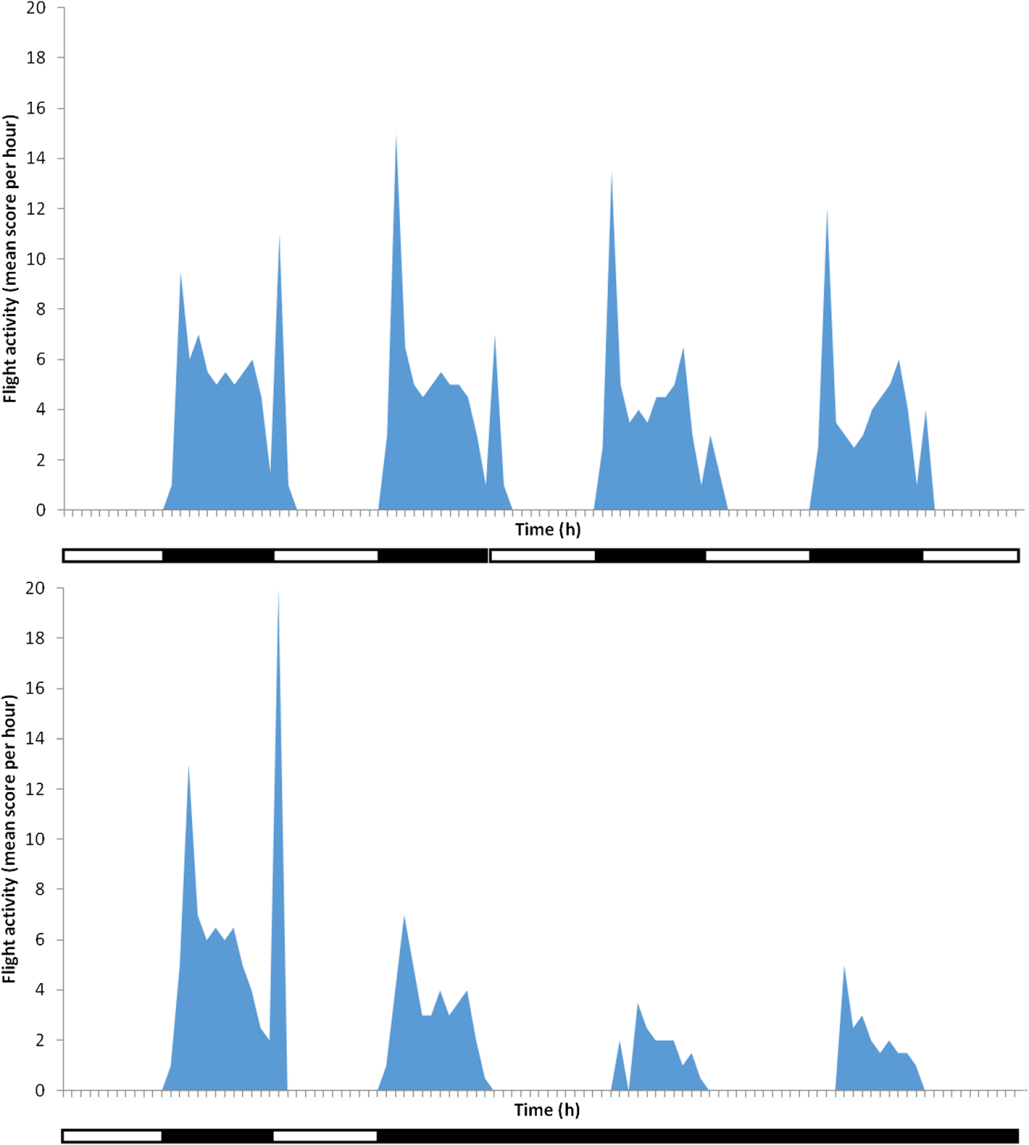
**Circadian flight activity of inseminated non blood-fed** ***Anopheles stephensi*** **in an acoustic actograph under a 12:12 hour light/dark regime (top) and on transfer from a light/dark 12:12 hour to a constant darkness regime (bottom).** Dark bars on x-axis refer to periods of darkness, white bars to periods of light. Hourly flight activity is a score (out of 60) indicating the number of minutes per hour during which mosquitoes undertook flight.

While *An. gambiae* responded to the toxic action of pyrethroid exposure by day and night, response to chlorfenapyr exposure was more evident in the night-time assay (tunnel test) than in the day-time assays (cone and cylinder). To explore this further the resting and flight activity of mosquitoes in chambers of similar size to cones was examined using an actograph to record spontaneous flight activity. In the LD 12:12 regime, sugar-fed inseminated females showed no activity during the 12-hour light phase but during the dark phase there was an activity peak shortly after light off, followed by short bursts of intermittent activity throughout the 12 hours of darkness and a small activity peak at ‘dawn’ as the dimmer switched from darkness to light (Figure 3). When, in the next experiment, the LD 12:12 regime was changed to constant darkness (DD 12:12), a peak of flight activity occurred regularly at 24-hour intervals during the period which coincided with the former dark phase but not the former light phase (Figure 3). Thus the activity observed in the dark phase of LD 12:12 was not a response to the switch from light to dark but the expression of a free-running circadian activity rhythm with a 24-hour periodicity that was being expressed during the dark phase of LD 12:12 cycles.

### Insecticide bioassay efficacy in relation to the phase of the mosquito circadian rhythm

During the first series in Tanzania, mean mortality induced by chlorfenapyr 200 mg/sq m samples tested at night was 92% after 24 hours and 100% after 72 hours (Table 1). The same samples tested during the day induced far lower levels of mortality, with a mean of 65% 24 hours after exposure (odds ratio = 8.5, 95% CI: 3.1-23.7, P <0.001, comparing night and day response). After 72 hours the difference in mortality between day-time and night-time exposure was less pronounced as mortality converged towards 100%. Whereas all samples tested at night scored 100% only one of the seven samples tested in the day-time reached 100%.

**Table 1:** **Comparison of day-time and night-time testing of chlorfenapyr 200 mg/sq m insecticide-treated net using 30-minute exposure in cylinder bioassays**

	**24 h Mortality**			**72 h Mortality**		
	**Day**	**Night**	**Odds ratio**	**Day**	**Night**	**Odds ratio**
**Tanzania,** ***An. gambiae*** **Kisumu**						
Chlorfenapyr 200 mg/sq m (95% CI)	**65** (56–72)	**92** (86–96)	**8.5** (3.1-23.7) P < 0.001	**90** (84–94)	**100**	**†**
**Tanzania,** ***An. arabiensis*** **F1**						
Chlorfenapyr 200 mg/sq m (95% CI)	**8** (5–12)	**41** (35–47)	**14.1** (5.9-33.6) P < 0.001	**26** (21–31)	**63** (57–68)	**10.5** (4.3-25.7) P < 0.001
**Benin,** ***An. gambiae*** **VKPER**						
Chlorfenapyr 200 mg/sq m (95% CI)	**9** (6–15)	**10** (6–16)	**1.0** (0.5-2.1) P < 0.974	**39** (32–47)	**58** (51–66)	**2.4** (1.5-4.0) P < 0.001

Notes: † indicates that statistical models could not produce an odds ratio estimate.

In the second series in Tanzania, chlorfenapyr 200 mg/sq m induced significantly greater mortality of *An. arabiensis* F1 when tested during the night than during the day (odds ratio 10.5, 95% CI 4.3-25.7, P <0.001) (Table 1). Mean 72-hour mortality was 26% (95% CI: 21–31) when tested during the day compared to 63% (95% CI: 57–68) at night. In the third series, tested in Benin, chlorfenapyr 200 mg/sq m produced a similar trend against the pyrethroid-resistant *An. gambiae* VKPER, scoring higher mortality when tested at night (mean 58%, 95% CI: 51–66) than when tested at day (mean 39%, 95% CI: 32–47) (odds ratio 2.4, 95% CI 1.5-4.0, P = 0.001) (Table 1).

### Effect of temperature on bioassay efficacy

**Figure 4:**
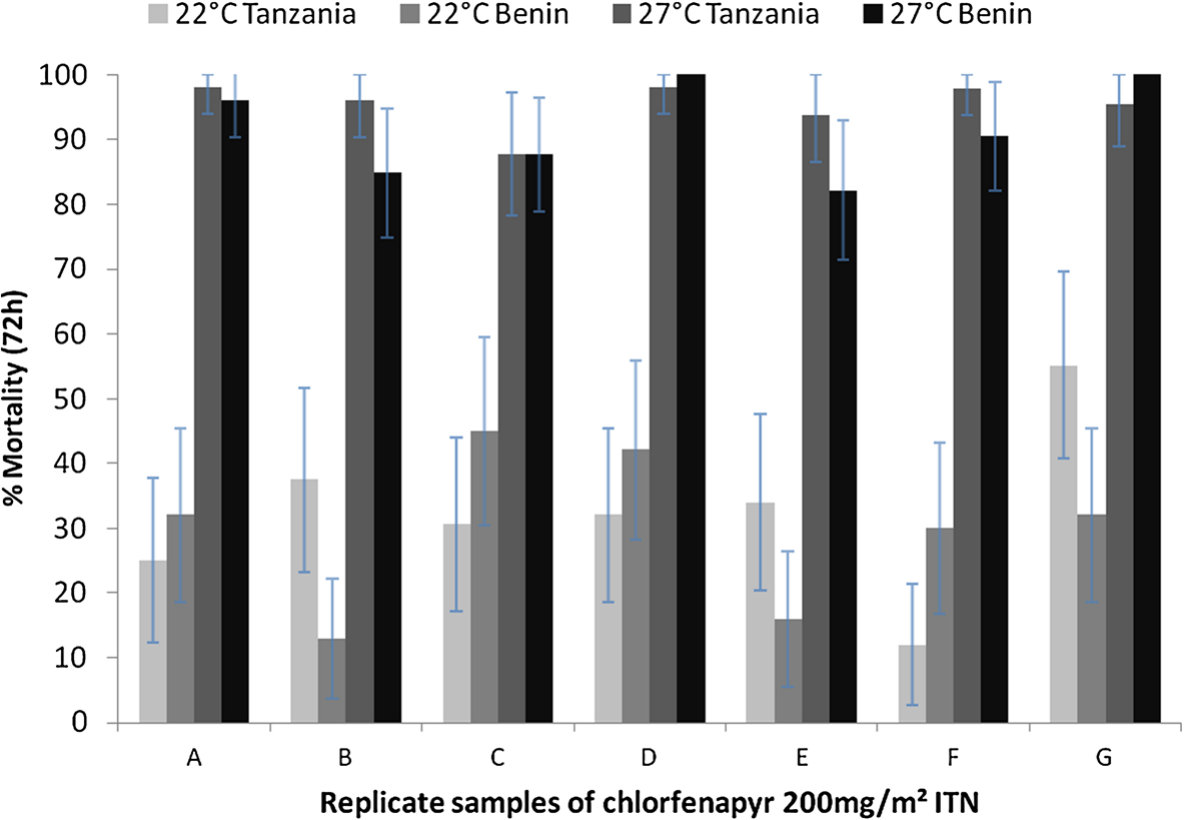
**Effect of temperature (22°C** ***versus*** **27°C) on % mortality (72 hours) in bioassays with** ***Anopheles gambiae*** **Kisumu tested on chlorfenapyr ITN in Tanzania and Benin after day-time exposure of 30 minutes in cylinder bioassays.**

In the first series the factor of interest on chlorfenapyr activity was temperature at 22°C or 27°C during testing and holding. The seven samples (A-G) treated with chlorfenapyr 200 mg/sq m killed between 12 and 45% when tested at 22°C and killed 82-100% when tested at 27°C (odds ratio 41, 95% CI: 27–63, P <0.001) (Figure 4). The country of testing had no significant effect (P = 0.154).

**Figure 5:**
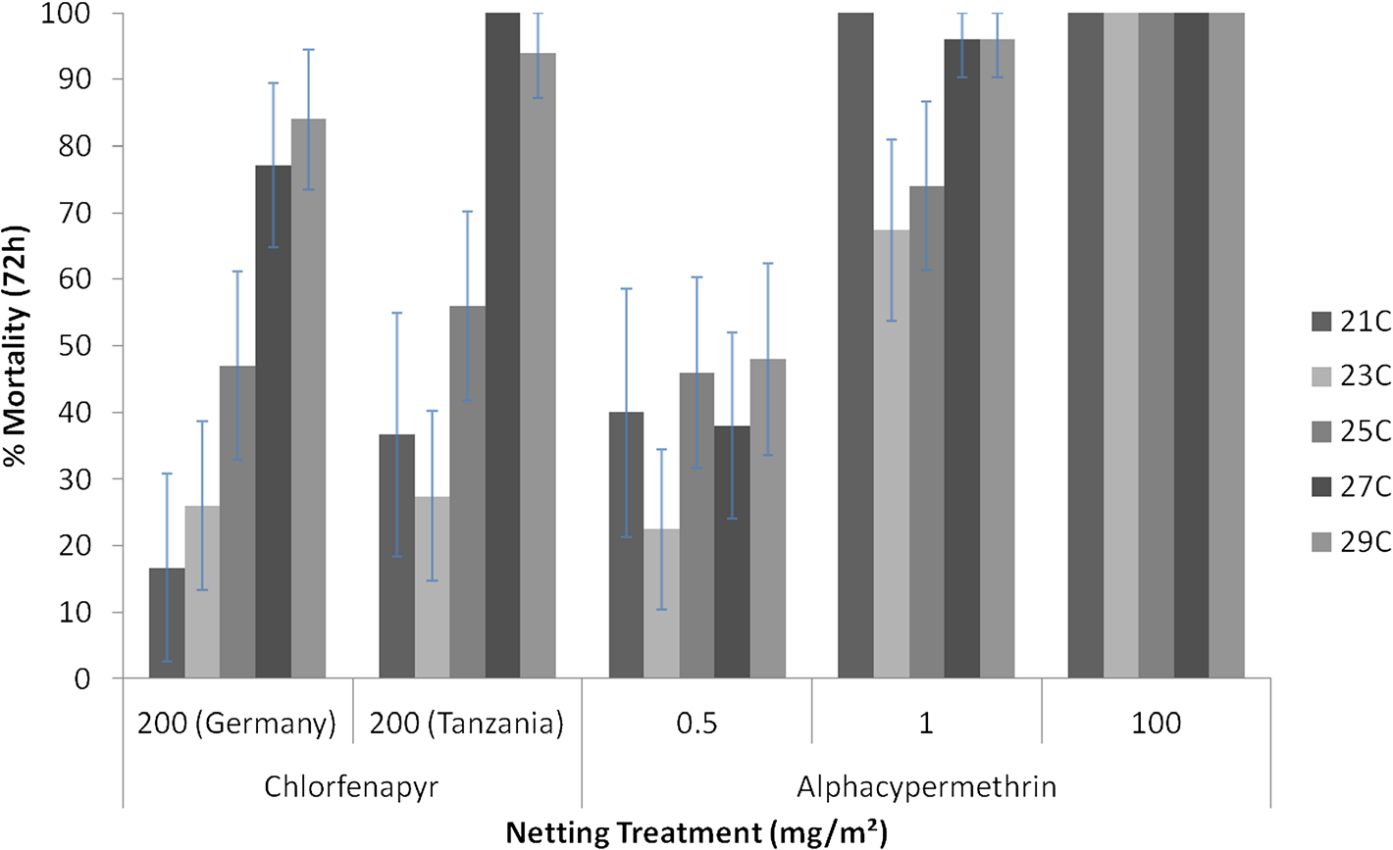
**Percentage mortality of** ***Anopheles gambiae*** **Kisumu (72 hours) following 30-minute cylinder bioassay of chlorfenapyr and alphacypermethrin ITN samples at 2°C intervals between 21and 29°C.**

**Figure 6:**
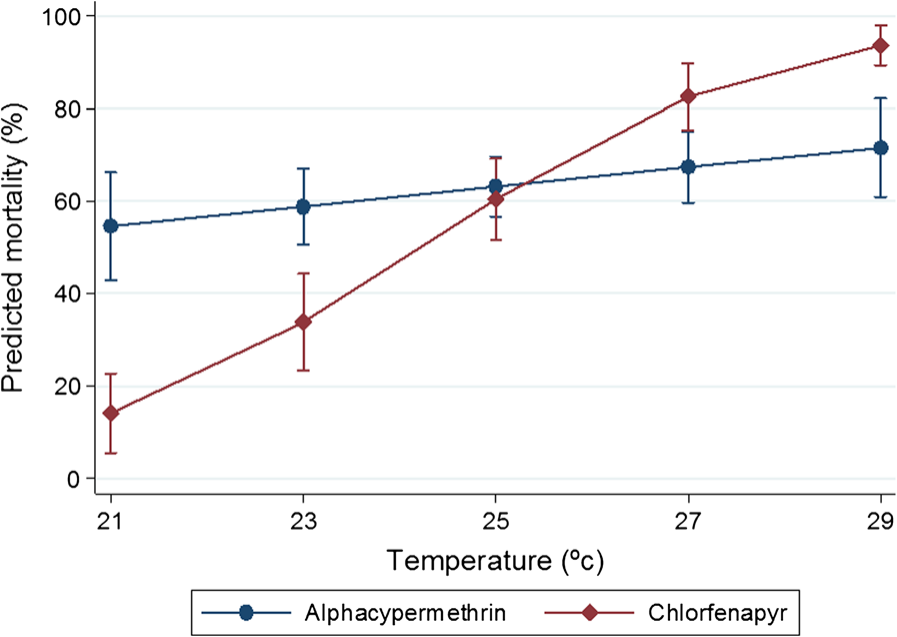
**Predicted mortality of** ***Anopheles gambiae*** **Kisumu by treatment between 21 and 29°C.**

In the second series, the bioassay mortality was compared at testing intervals of 2°C in a range of 21-29°C. Chlorfenapyr 200 mg/sq m samples showed a strong positive temperature coefficient, with mortality increasing with every increment of 2°C. Focusing on the WHOPES recommended testing range of 27°C ± 2°C, there was an odds ratio of 10.4 (95% CI = 5.5-19.6, P <0.001) associated with the 4°C increase in temperature from 25-29°C for chlorfenapyr ITN compared with only 1.7 for alphacypermethrin (95% CI = 0.9-3.1, P = 0.075). The alphacypermethrin net of 100 mg/sq m killed 100% of pyrethroid-susceptible *An. gambiae* Kisumu at all temperatures. To improve the prospect of discriminating between temperature intervals, lower dosages of alphacypermethrin at 0.5 and 1 mg/sq m were tested. While this had the desired effect of killing less than 100%, the proportions killed across the 21-29°C range were similar at all intervals (Figure 5). Predicted mean mortality projections for chlorfenapyr showed strong evidence (odds ratio = 1.8; 95% CI 1.5-2.1, P <0.001) of a mortality-temperature response for every 1°C increase in temperature. There was little evidence of a mortality gradient with temperature for alphacypermethrin (odds ratio = 1.1; 95% CI 1.0-1.3, P = 0.075) (Figure 6).

## Discussion

Pyrethroid resistance has spread rapidly as a result of scaling up LLIN and indoor residual spraying (IRS) and is now present at high frequency in many areas of sub-Saharan Africa [2]. It is clear that new insecticides for LLIN are urgently needed if momentum towards malaria elimination is to continue [1]. Pyrethroid insecticides have ideal properties for use on mosquito nets. They are highly toxic and fast acting against mosquitoes, provide repellency and personal protection [24,25], are safe for users (low mammalian toxicity) [26], and can be readily made into wash-resistant LLINs [4]. New insecticides are unlikely to have the same properties of rapid knock-down and mortality but can still be effective in transmission control if mosquitoes contact treated netting for a sufficient duration.

There is limited information on how long mosquitoes spend in contact with untreated or treated mosquito nets. Hossain and Curtis used artificial releases to demonstrate that susceptible *An. gambiae* spend up to 21 minutes in contact with an untreated net but only three minutes on a permethrin-treated net [27]. Three minutes is the standard WHO specified cone bioassay exposure time for ITNs regardless of the insecticide evaluated. This is a suitable duration of exposure for neurotoxic, excito-repellent, pyrethroid insecticides, where a mosquito either picks up a lethal dose or is repelled within a short time of contacting the ITN. Other classes of insecticide may require longer exposures to produce high levels of mortality. The time spent in contact with the net is influenced by the contact-irritancy of the insecticide [28]. Once mosquitoes become resistant to pyrethroids they show less irritability and spend longer in contact with the net [28]. Calibration of exposure time and mortality in bioassay with efficacy of alphacypermethrin-treated nets in experimental huts indicates that pyrethroid-resistant *An. arabiensis* spends much longer than three minutes but less than 30 minutes in contact with the treated net. The calibration of chlorfenapyr bioassay results with experimental hut efficacy justifies longer exposure times for non-repellent insecticides in WHO cone bioassay tests.

WHOPES sets the international standards for testing LLIN [8]. WHOPES guidelines state that “The efficacy of treated nets may be underestimated if judged based on the outcome of standard cone bioassays” [8]. If a candidate LLIN fails to achieve the threshold level of mortality in a three-minute cone test (80% mortality), as sometimes happens with more repellent pyrethroids, its efficacy is evaluated in the overnight tunnel test [8]. In the laboratory comparison of alphacypermethrin and chlorfenapyr, the pyrethroid easily met the threshold mortality in the cone test and did not require to go forward to tunnel testing. Chlorfenapyr produces little or no irritancy at application rates <500 mg/sq m [11,29], and yet in the cone and cylinder tests not only did chlorfenapyr fail to achieve the threshold 80% mortality with three-minute exposure, it could only achieve half this level of mortality with 30-minute exposure. Only the laboratory tunnel test achieved a level of mortality that met the 80% threshold [8].

How is it that an insecticide that failed the cone criteria so abjectly can produce excellent results in overnight tunnel tests and experimental huts? The answer resides in the unique mode of action. Unlike neurotoxic pyrethroids, chlorfenapyr acts by disrupting metabolic respiratory pathways (oxidative phosphorylation) in the mitochondria of cells [10]. It is also a pro-insecticide requiring conversion to the active compound by the action of cellular mixed-function oxidases [10]. The expression in *An. gambiae* of some cytochrome P450s involved in oxidative metabolism are under circadian control and more strongly expressed at night [30]. Anopheline flight and host-seeking activity is a high-energy, high-respiratory behaviour which is also under circadian control and occurring only at night [20,21]. A model for chlorfenapyr is that the metabolic conversion to the active compound, CL 303268, is more strongly expressed at night, at the time of day when cellular respiration is also at its zenith due to the circadian flight activity rhythm and when the uncoupling of respiratory pathways would be at their most disruptive to the mosquito [20,21]. Such a model would explain the high levels of mortality in experimental huts and night-time bioassays against active mosquitoes (tunnel test and night-conducted cylinder test) and also account for the low level mortality observed in day-time bioassays.

The activation of chlorfenapyr and its toxic action of disrupting cellular respiration, being metabolic processes, are both presumed to be temperature dependent. This would explain the stronger correlation between temperature and day-time bioassay mortality with chlorfenapyr than observed with the pyrethroid alphacypermethrin. Crucial to the toxicity of chlorfenapyr is the circadian rhythm of the mosquito. During the night, when mosquitoes are at their most behaviourally active due to their circadian phase, exposure to chlorfenapyr induces a high level of mortality independent of ambient temperature. This concurs with the high mortality (53.5%) observed during hut trials [18] in equatorial Benin (altitude 9 m) where average temperature was 27°C and the similarly high mortality (48%) during trials [19,22] in upland areas of Moshi, Tanzania (altitude 760 m) where average temperature was only 23°C and the average night-time temperature of 17°C was 8°C lower than the 25°C of Benin [31]. Presumably the circadian phase, or the expression of chlorfenapyr toxicity on cellular respiration during that phase, has the capacity to offset or over-ride any effect of reduced ambient temperature. Because wild, host-seeking *Anopheles* are more metabolically active than mosquitoes in day-time bioassay, this can mask the effect of low ambient temperature in highland areas, as indicated in the published trials with chlorfenapyr ITNs and IRS in cooler Moshi, Tanzania [18,19,22]. In short, chlorfenapyr is effective both in lowland tropical locations where night temperatures are particularly hot and in highland areas where temperatures are cooler.

Circadian rhythm and ambient temperature both affect metabolic status. Most types of laboratory bioassays to determine the efficacy and wash-resistance of LLIN are conducted during day time [8].The model to explain the improved performance of chlorfenapyr ITN when tested at night can also explain the improved day-time mortality when a mosquito is more metabolically active due to raised ambient temperature. WHOPES guidelines for evaluation of LLINs state that contact bioassays should be conducted at 27°C ± 2°C (i.e., 25-29°C) [8]. Considering the high odds (10.4) for *An. gambiae* mortality with chlorfenapyr ITN at 29°C compared to 25°C, this is likely to lead to significant variation in test results between laboratories unless strict temperature control, such as the use of incubators and data loggers are deployed as quality assured best practice. Bioassay testing temperature is less sensitive for pyrethroids. In this study there was no significant evidence of a temperature-mortality gradient for alphacypermethrin. Generally for pyrethroids a negative temperature coefficient with mortality has been recorded for the majority of insect species evaluated, over a larger temperature range [32-35], and appears to be due to greater nerve sensitivity [32].

Because experimental hut trials simulate domestic conditions, they provide the definitive test of LLIN efficacy [8]. Cone or cylinder bioassays conducted at night, when the phase of the circadian rhythm means that that the mosquito is both behaviourally and metabolically active, are likely to be more predictive of efficacy in huts. Such testing is inconvenient to perform unless mosquito rearing and testing are carried out under insectary reverse photophase. The overnight tunnel test is likely to be the more accurate predictor of field performance as the mosquito is host seeking during the active phase of the circadian rhythm, and contacts netting in a more realistic way when attempting to reach the animal host. Despite being technically more demanding, overnight tunnel tests should always be conducted when screening for insecticidal activity from novel classes to ensure that insecticides which may be potent when tested against wild host-seeking mosquitoes are not overlooked based on an artificial, fixed exposure bioassay.

WHOPES guidelines have been developed for the evaluation of pyrethroid nets [7,8]. New insecticides for LLIN, such as chlorfenapyr, will not have the same properties as pyrethroids; ultimately, high mortality and low blood feeding in field trials against wild malaria vectors are the most important measures of success. These studies have highlighted the need to adapt laboratory-testing protocols for the evaluation of novel, non-neurotoxic insecticides. If current WHOPES guidelines were to be rigidly followed, there is a danger that insecticides that are highly effective against wild mosquitoes, such as chlorfenapyr, would be overlooked at the screening stage of evaluation through bioassay and never progress to field evaluation. The current emphasis on Phase 1 test criteria and thresholds developed and tailored for pyrethroids will not serve for the new classes of insecticide. Revision of the WHOPES LLIN evaluation guidelines, putting emphasis on tunnel tests which simulate or allow expression of night-phase, host-seeking behaviour on the net, are urgently required.

## Conclusions

The pyrrole insecticide chlorfenapyr shows great promise as an alternative to pyrethroids for use on nets. Its mode of action and characteristics in laboratory bioassay differ from those of neurotoxic insecticides such as pyrethroids. Standard WHO bioassay test criteria (such as the 80% mortality threshold) may not be achievable and such tests may fail to predict the performance of novel active ingredients in field trials. The three-minute exposure cone test should be abandoned as screening bioassay for novel insecticides because the exposure time is too short. Exposure time for novel active ingredients should be established by calibration of mortality of free-flying mosquitoes in experimental huts with bioassay mortality across a range of exposure times. For non-neurotoxic insecticides, which act or disrupt metabolic pathways, the bioassay should take into account the mosquito’s circadian activity rhythm and metabolic status. The WHO tunnel test on night-active mosquitoes is the most relevant and predictive bioassay for identifying or establishing the toxicity of new active ingredients on nets.
